# Small molecule CP-31398 induces reactive oxygen species-dependent apoptosis in human multiple myeloma

**DOI:** 10.18632/oncotarget.19508

**Published:** 2017-07-22

**Authors:** Yohei Arihara, Kohichi Takada, Yusuke Kamihara, Naotaka Hayasaka, Hajime Nakamura, Kazuyuki Murase, Hiroshi Ikeda, Satoshi Iyama, Tsutomu Sato, Koji Miyanishi, Masayoshi Kobune, Junji Kato

**Affiliations:** ^1^ Department of Medical Oncology, Sapporo Medical University School of Medicine, Sapporo, Japan; ^2^ Department of Hematology, Sapporo Medical University School of Medicine, Sapporo, Japan

**Keywords:** CP-31398, ROS, apoptosis, multiple myeloma

## Abstract

Reactive oxygen species (ROS) are normal byproducts of a wide variety of cellular processes. ROS have dual functional roles in cancer cell pathophysiology. At low to moderate levels, ROS act as signaling transducers to activate cell proliferation, migration, invasion, and angiogenesis. In contrast, high levels of ROS induce cell death. In multiple myeloma (MM), ROS overproduction is the trigger for apoptosis induced by several anticancer compounds, including proteasome inhibitors. However, no drugs for which oxidative stress is the main mechanism of action are currently used for treatment of MM in clinical situations. In this study, we demonstrate that the p53-activating small molecule CP-31398 (CP) effectively inhibits the growth of MM cell lines and primary MM isolates from patients. CP also suppresses the growth of MM xenografts in mice. Mechanistically, CP was found to induce intrinsic apoptosis in MM cells via increasing ROS production. Interestingly, CP-induced apoptosis occurs regardless of the p53 status, suggesting that CP has additional mechanisms of action. Our findings thus indicate that CP could be an attractive candidate for treatment of MM patients harboring p53 abnormalities; this satisfies an unmet clinical need, as such individuals currently have a poor prognosis.

## INTRODUCTION

Reactive oxygen species (ROS) are normal byproducts of a wide variety of cellular processes, including oxygen metabolism [1, 2]. Accumulating evidence suggests that ROS have ‘double-edged sword’ activity in cancer cells. At low to moderate levels, ROS act as signaling transducers to activate cell proliferation, invasiveness, angiogenesis, and drug resistance [1, 3, 4]. Indeed, we previously reported that induction of ROS by excess intracellular iron evoked malignant progression in multiple myeloma (MM) cells [5]. In contrast, high levels of ROS induce cell damage and ultimately lead to cell death [6]. Several compounds that are approved as MM therapeutics induce apoptosis via increasing intracellular ROS levels [7, 8]. Thus, elevating ROS levels is a valid strategy for combating MM.

MM is an incurable malignancy characterized by the expansion of plasma cells in the bone marrow. Novel therapeutic agents including proteasome inhibitors, immunomodulatory drugs (iMIDs), and histone deacetylase inhibitors have extended overall survival for patients with MM [9]. However, subsets of patients become refractory to treatment, underscoring the need for more effective therapeutic options.

p53 is a critical tumor suppressor gene that is mutated or deleted in approximately 50% of human cancers. In MM, p53 status is an independent prognostic marker, since patients harboring p53 abnormalities are highly resistant to standard therapies and incidences of p53 mutations and deletions increase during disease progression [10–12]. Therefore, restoration of p53 is an attractive strategy for advanced relapsed and refractory MM (RRMM) patients.

CP-31398 (CP) is a small molecule that activates wild-type p53 or restores tumor-associated p53 mutants to wild type p53 function in multiple human cancer cell lines; this leads to cell cycle arrest and/or apoptosis [13, 14]. The growth of rhabdomyosarcoma (RMS) cell lines can be inhibited by p53-dependent induction of ROS [15]. However, whether CP-induced cytotoxicity proceeds via a similar pathway is currently not investigated in MM.

In the present study, we demonstrate that CP exerts anti-myeloma activity regardless of p53 status both *in vitro* and *in vivo* settings. CP induced the generation of mitochondrial and cytosolic ROS, depolarization of mitochondrial membrane potential, and subsequent caspase-dependent apoptosis in MM cells. These findings suggest that CP is an effective ROS inducer irrespective of p53 status, and therefore suggest that CP could be used to treat RRMM even in patients harboring p53 abnormalities.

## RESULTS

### CP inhibits proliferation of MM cells *in vitro*

**Figure 1:**
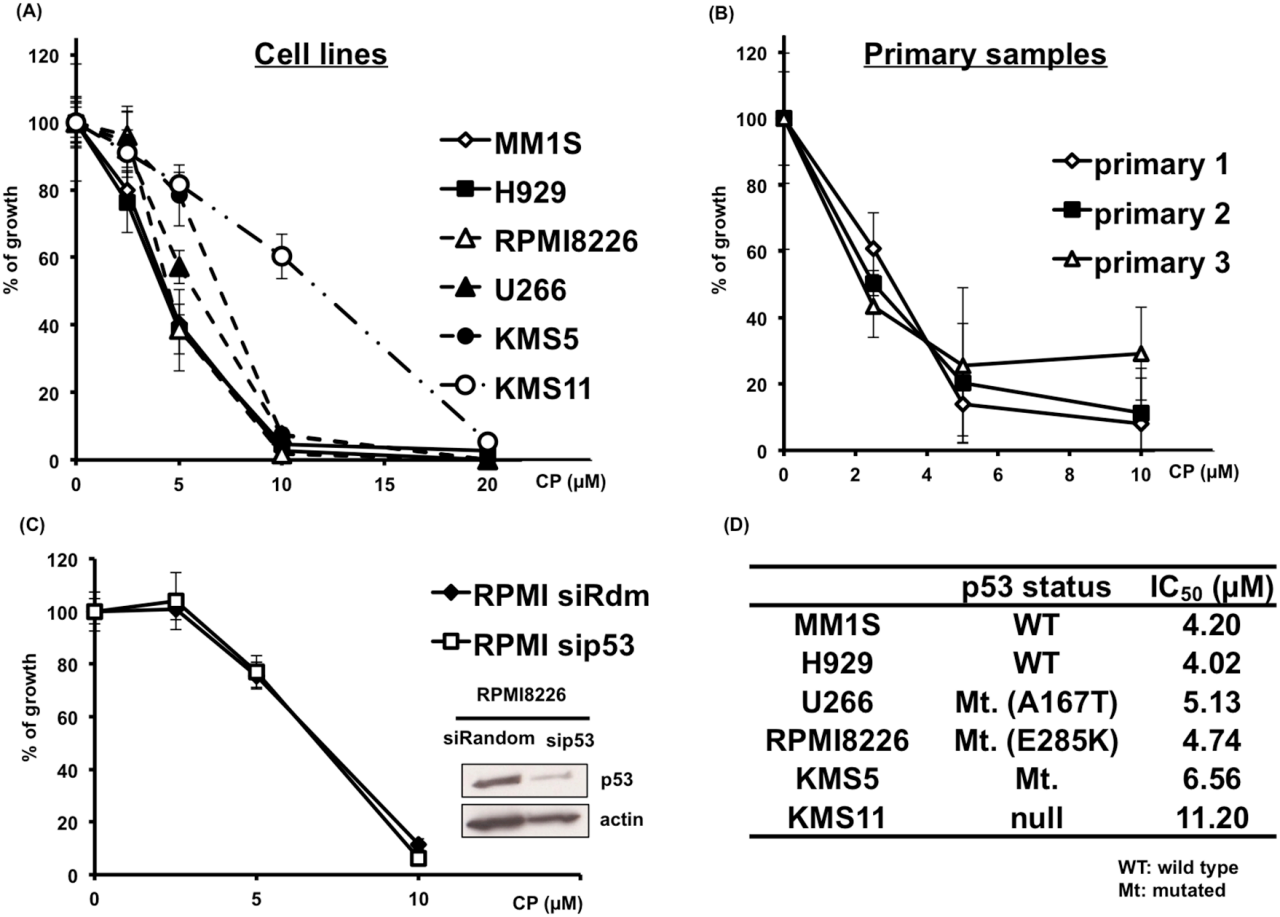
CP inhibits the proliferation of cultured MM cells **(A, D)** MM cell lines expressing wild-type p53 (MM1S, H929), mutant p53 (RPMI8226, U266, KMS5) or p53 null (KMS-11) were cultured with CP (0–20 μM). Cell viability was determined at 48 h using a WST-1 assay. **(B)** CP induced cytotoxicity in primary MM samples. Cell viability was expressed as percentage of the DMSO-treated control at 48 h using a WST-1 assay. **(C)** siRandom or sip53 transfected RPMI8226 cells were incubated with CP for 48 h and cell viability was assessed using a WST-1 assay. Mt.: Mutated, WT: wild type. Data represents means of 6 independent cultures. Error bars represent the SD. *P < 0.01.

Because no previous report has confirmed the anti-myeloma activity of CP, we first verified the cytotoxicity of CP in MM cells. CP significantly reduced the proliferation of MM cells in a dose-dependent manner regardless of their p53 status (Figure 1A and 1C). Interestingly, CP showed cytotoxicity even for p53-silenced RPMI8226 cells (Figure 1C) or KMS11 as p53-null cells (Figure 1A). A significant decrease in viability of these samples was observed, with IC_50_ values in the range of 4.02–11.2 μM (Figure 1D). Notably, CP inhibited the proliferation of MM cells concentrations lower than those required for a similar effect in cancer cell lines derived from other tumor types [14–18]. To further assess the anti-myeloma potential of CP, primary MM samples derived from three newly diagnosed MM patients were treated with the compound (Figure 1B) and CP showed a similar anti-myeloma effect for primary MM cells. None of the primary samples harbored del17p.

Since bone marrow stromal cells (BMSCs) promote MM cell proliferation and induce drug resistance, we tested the effect of BMSCs on the sensitivity of MM cells to CP treatment using BrdU assays. CP maintained its anti-myeloma activity even in the presence of BMSCs (Supplementary Figure 1).

### CP induces apoptosis in MM cells

**Figure 2:**
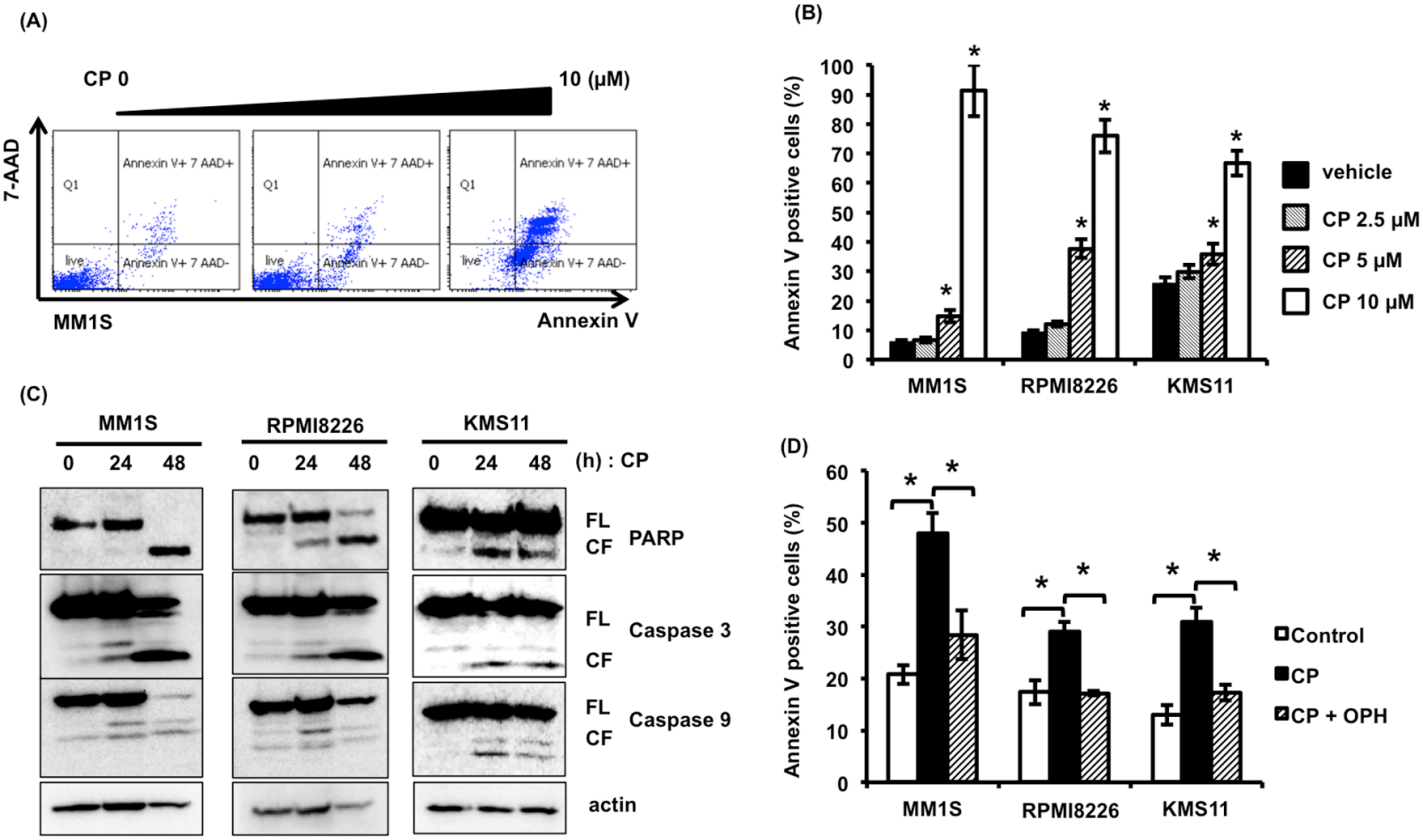
CP induces apoptosis of MM cells regardless of their p53 status **(A, B)** MM cells were treated with CP (0–10 μM) for 24 h. Apoptosis was measured by flow cytometry using Annexin V/7-AAD staining. Data are the mean of triplicate measurements. Error bars represent the SD. *P < 0.01. **(C)** MM cell lines were treated with the IC_50_ dosage of CP for 0, 24, and 48 h. Whole cell lysates were subjected to immunoblotting using anti-caspase 9, caspase 3, PARP, and beta-actin antibodies. FL; full length, CL; cleaved form. **(D)** MM cells were treated with the IC_50_ dosage of CP with or without pan-caspase inhibitor Q-VD-OPH (OPH; 20 μM). Q-VD-OPH was pre-added 1 h before CP treatment. Apoptosis was measured by flow cytometry using Annexin V/7-AAD staining. *P < 0.01.

To clarify the mechanism of CP-induced cytotoxicity in MM cells, we examined the proteins involved in programmed cell death by immunoblotting and performed flow cytometry analysis. As shown in Figure 2A and 2B, Annexin V/7-AAD staining showed an increased percentage of apoptotic cells treated with CP. Additionally, CP increased proteolytic cleavage of caspase-9, caspase-3, and PARP, but not caspase-8 (data not shown) in MM1S (p53 wild type), RPMI8226 (p53 mutated), and KMS11 (p53 null) cell lines (Figure 2C). Besides, co-treatment with the pan-caspase inhibitor, Q-VD-OPH, inhibited CP-induced cytotoxicity (Figure 2D). These data indicate that CP-induced cytotoxicity in MM cells is mediated via caspase-dependent intrinsic apoptosis.

**Figure 3:**
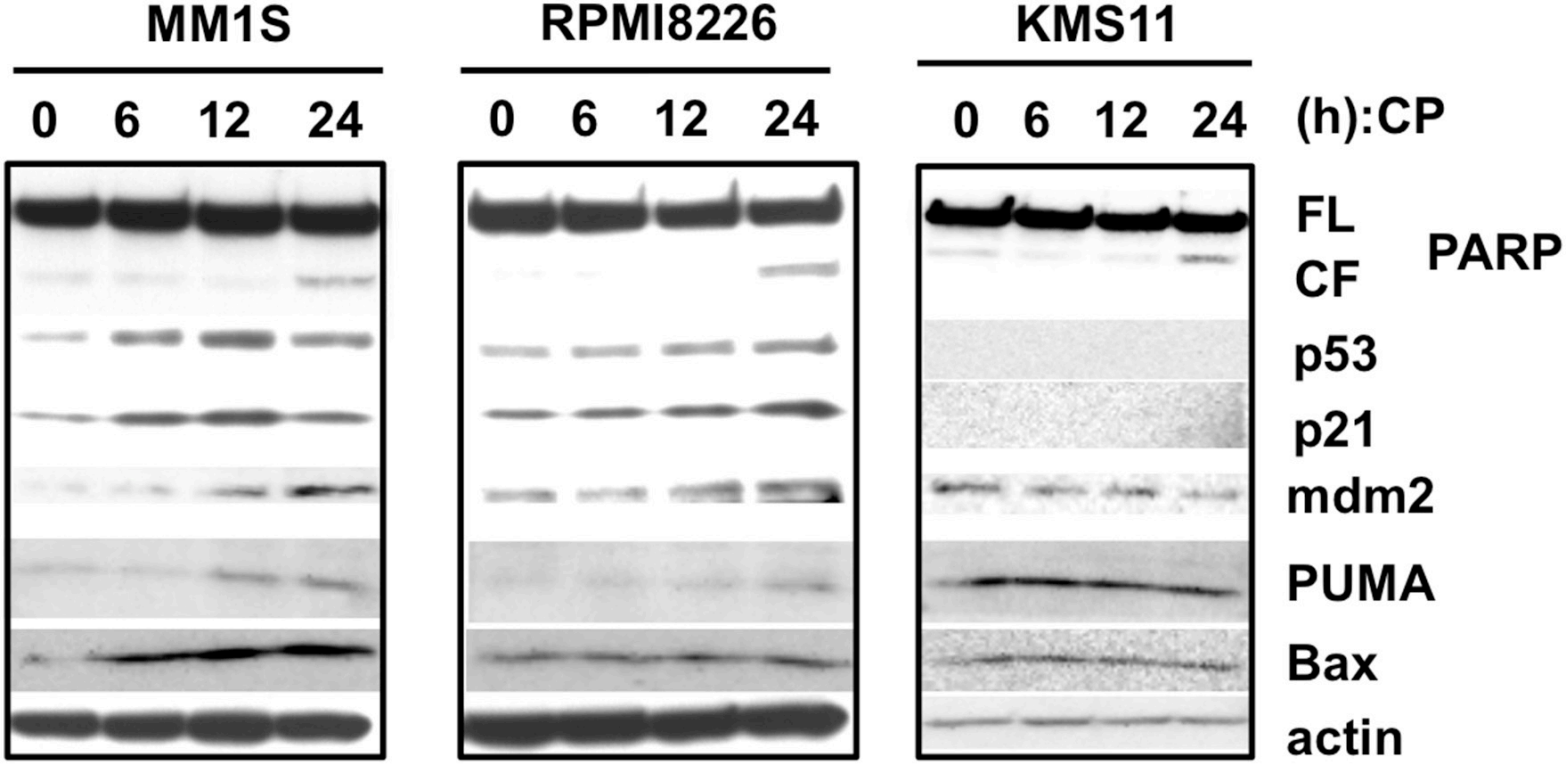
CP treatment slightly increase the levels of p53 and its downstream targets MM cell lines were treated with the IC_50_ dosage of CP for 0, 6,12, and 24 h. Whole cell lysates were subjected to immunoblotting using anti-PARP, p53, p21, mdm2, PUMA, Bax, and beta-actin antibodies. FL; full length, CL; cleaved form.

Because CP increases the level and activity of wild-type p53 and can also restore wild type function to mutated p53 proteins in many cancer cells, we evaluated the p53 pathway in MM cells exposed to the drug. Increased levels of p53 and its downstream targets, including p21, mdm2, and PUMA were observed to some extent in MM1S and RPMI8226 (Figure 3). However, p53-silenced MM cells remained sensitive to CP (Figure 1C, Supplementary Figure 2). Together with our findings that CP was also cytotoxic in p53-null KMS11 cells, these results suggest that CP, which is known as a “p53-restoring molecule”, has p53-independent mechanisms of action. In other words, p53 is not required for CP-induced cytotoxicity in MM cells.

### CP induces ROS-dependent apoptosis

**Figure 4:**
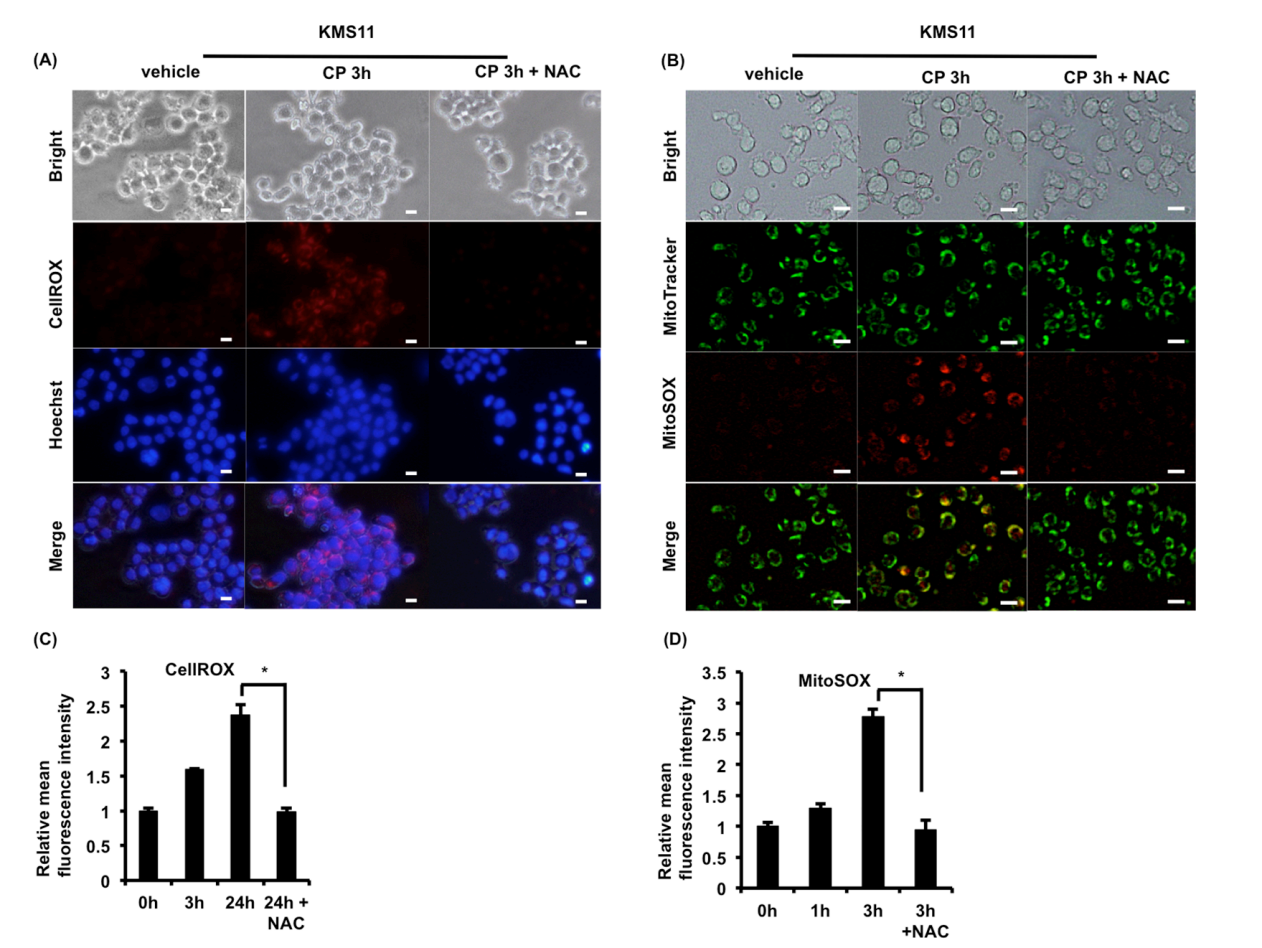
CP treatment stimulates the generation of cytosolic and mitochondrial ROS in MM cells **(A, B)** KMS11 cells were cultured with CP (10 μM) in the absence or presence of NAC (10 mM, 2 h) pretreatment. Thereafter, cytosolic and mitochondrial ROS of KMS11 were monitored by live cell microscopy using CellROX and MitSOX, respectively. Scale bars, 10 μm. **(C, D)** Cytosolic and mitochondrial ROS levels in CP-treated RPMI8226 (CP, 5 μM) were measured by flow cytometry. Data are the mean of triplicate measurements. Error bars represent the SD. *P < 0.01.

ROS play an important role in MM, and CP was reported to induce ROS in RMS cells [15]. Therefore, we next measured intracellular and mitochondrial ROS levels to investigate whether CP stimulated ROS generation in MM cells. Following treatment of RPMI8226 and KMS11 cells with CP, an increased generation of intracellular and mitochondrial ROS was observed (Figure 4A-4D). Moreover, the ROS scavenger N-acetyl-cysteine (NAC) markedly decreased the level of ROS induced by CP.

**Figure 5:**
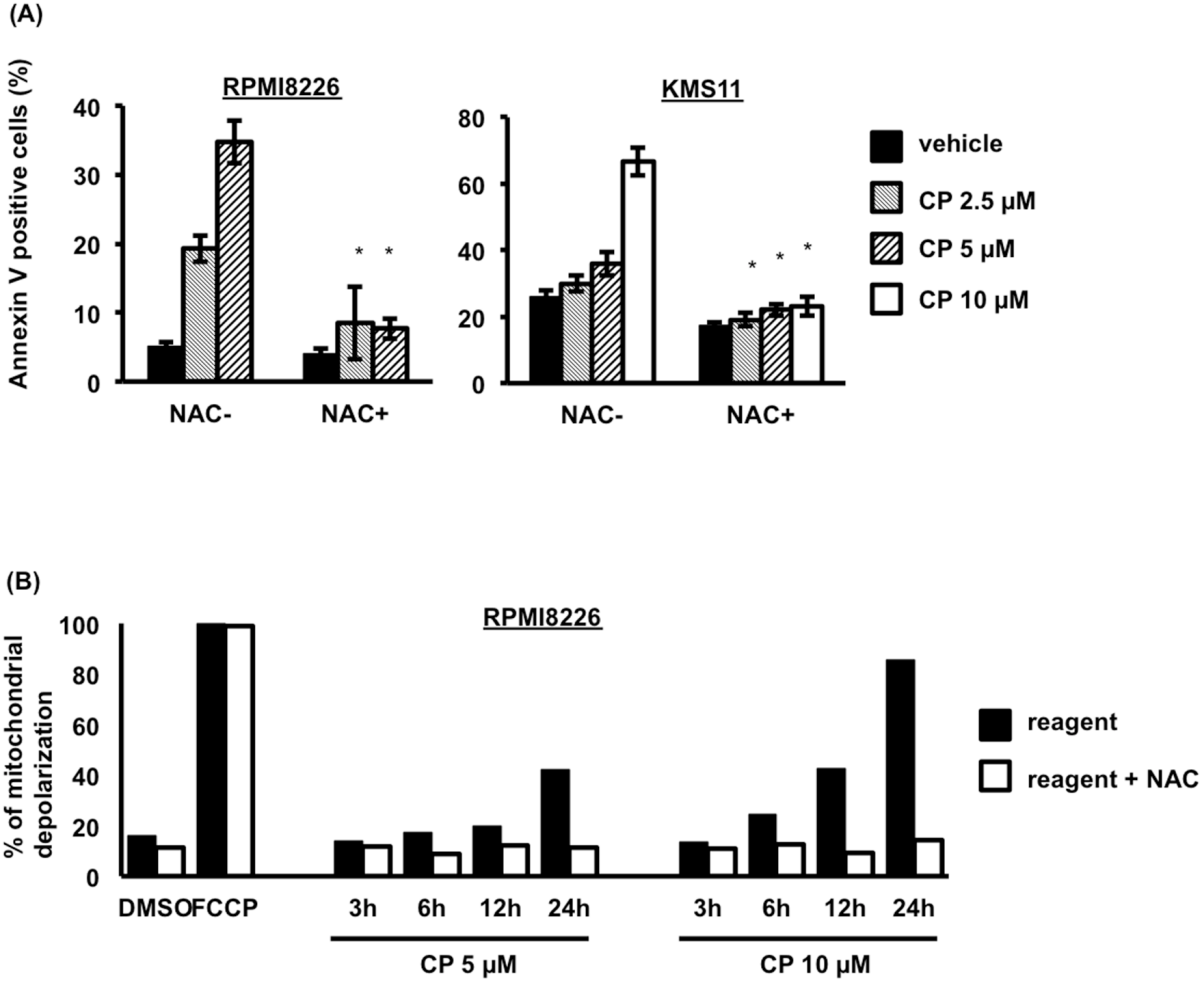
CP induces ROS-dependent apoptosis of MM cells **(A)** Pretreatment with NAC (10 mM, 2 h) blocked CP-induced apoptosis in RPMI8226 and KMS11 cells. Data are the mean of triplicate measurements. Error bars represent the SD. *P < 0.01. **(B)** Effect of CP on the mitochondrial Δψm of RPMI8226 cells. Cells treated with CP for 0–24 h were stained with TMRE and measured using flow cytometry. The percentage loss of Δψm in the CP treated cells was calculated. Δψm, membrane potential.

Interestingly, NAC treatment dramatically decreased the percentage of apoptotic cells following CP treatment (Figure 5A), suggesting that ROS were the major determinants of apoptosis induced by the compound in MM cells. Consistent with a role for ROS in CP-induced death, mitochondrial membrane depolarization was prevented by NAC pretreatment (Figure 5B).

We next screened a panel of human oxidative stress markers using a PCR array to gain insight into the source of ROS following CP treatment in MM cells. This revealed a CP-dependent increase in the expression of oxidative stress-responsive genes (Supplementary Figure 3). However, the molecular mechanism underlying how CP induces ROS in MM cells was not elucidated.

### Anti-tumor activity of CP in MM xenograft models

**Figure 6:**
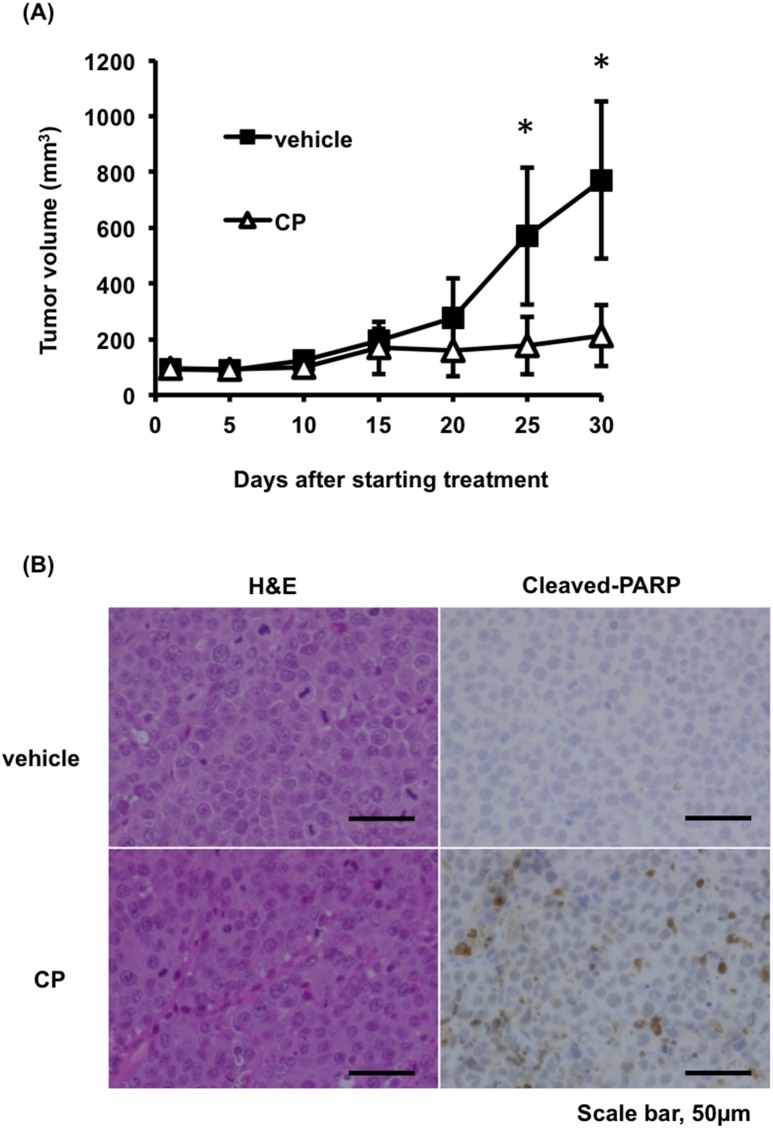
CP inhibits tumor growth in a mouse xenograft model of MM **(A)** Tumor volumes were calculated from caliper measurements. Data represent mean ± SD (n = 5). **(B)** Histologic analysis of tumors stained with hematoxylin and eosin (H&E) and cleaved-PARP antibody isolated from vehicle or CP-treated mice. Scale bars, 50 μm. *P < 0.01.

To evaluate the possibility of the clinical use of CP, we assessed its ability to suppress tumor growth *in vivo* using a subcutaneous RPMI8226 murine xenograft model. As shown in Figure 6A, CP treatment significantly reduced the MM tumor burden when compared to vehicle-treated controls. We also detected an increase in apoptotic tumor cells in mice treated with CP as determined by cleaved PARP staining (Figure 6B). CP treatment for one month was well tolerated and was not associated with loss of body weight (Supplementary Figure 4). Upon necropsy, no histologic changes in normal tissues were observed in any animal in the treatment group (Supplementary Figure 5). Collectively, these data provided evidence of the possibility of the clinical application of CP in MM.

### CP combined with proteasome inhibitor or histone deacetylase inhibitor inhibits the proliferation of cultured MM cells

**Figure 7:**
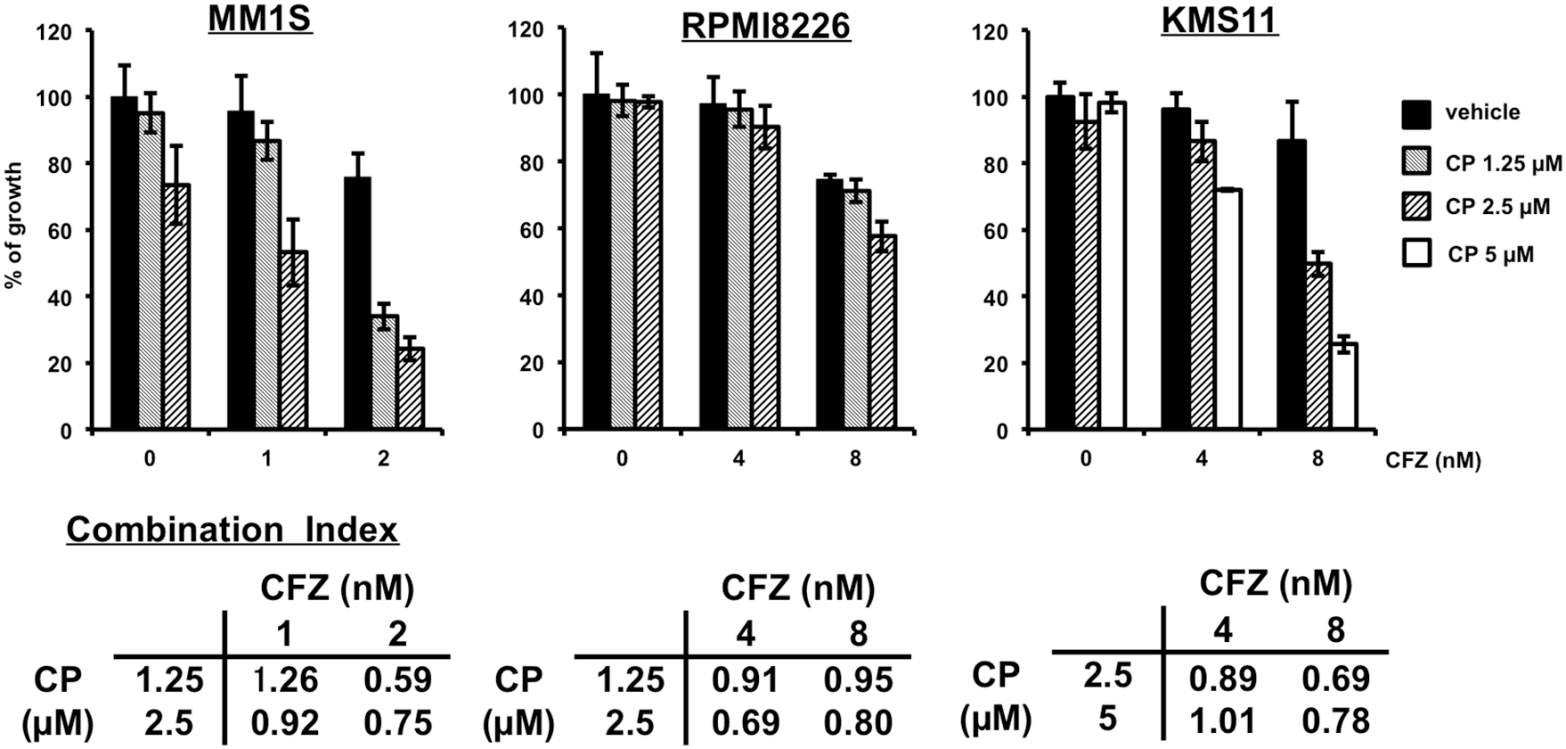
CP combined with CFZ inhibits the proliferation of cultured MM cells synergistically MM1S, RPMI8226 or KMS11 cells were incubated for 48 h with CP and CFZ. Thereafter, cell viability was assessed using a WST-1 assay. Data represent means of 6 independent cultures. Error bars represent the SD. Combination Index; CI. CI <1; synergetic, CI=1; additive effect.

To test whether CP might synergize with existing MM therapeutics, we examined the following two treatment combinations. First, we found that the cytotoxic effects of CP on MM cells were enhanced by the protease inhibitor carfilzomib (CFZ) (Figure 7). As for a combination with another class of drug, we studied combination treatment with the pan-histone deacetylase inhibitor, suberoylanilide hydroxamic acid (SAHA). CP showed low synergy even with SAHA (Supplementary Figure 6). Taken together, these combination therapies might be useful for MM patients in a clinical setting.

## DISCUSSION

In the current study, our data demonstrated that CP dramatically induced apoptosis in MM cell lines regardless of their p53 status. Of note, CP-dependent suppression of tumor growth in a murine xenograft model was not associated with any severe toxicity in normal tissues. In addition, it was reported that hematologic toxicity was not very significant in CP treatment [19], suggesting its potential clinical utility for MM treatment.

Accumulated evidence showed that CP has anti-tumor effects in a subset of cancers [14, 15]. This is the first report demonstrating the anti-myeloma effect of CP. However, in the current study we did not fully elucidate the mechanism of CP-driven cytotoxicity. According to previous studies, CP exerts an anti-proliferative effect in cancer cells through restoring tumor suppressive functions to mutant p53 and/or by activation of wild type p53-dependent transcription [13, 16]. CP can also induce translocation of p53 to mitochondria, leading to mitochondrial membrane depolarization, increased ROS production, and apoptosis in RMS cells [15]. Contrary to those findings, we observed only slight upregulation of p53 target genes after CP treatment and found that CP robustly induced apoptosis even in p53-null cells and in those in which p53 was silenced by siRNA. Interestingly, the cell-permeable antioxidant NAC substantially reversed CP-induced apoptosis, suggesting that this cell death was ROS-dependent. Although our PCR array analysis revealed clear increases in expression of genes induced by oxidative stress, the precise mechanisms underlying CP-dependent induction of ROS-dependent apoptosis in MM remain unclear. Further investigations in this area are therefore required.

BMSCs play a critical role in MM cell growth and chemoprotective effects [20]. We showed that CP suppressed MM cell growth with BMSCs. However, the cytotoxic effects of CP were reduced under the conditions employed. A previous study demonstrated that BMSCs inhibited apoptosis induced by PRIMA-1^Met^, which is a p53-restoring molecule, through a decrease in ROS production [21]. Therefore, the plausible mechanism for this effect was as follows: BMSCs interfered with CP-triggered apoptosis via decreasing ROS production.

Recently, Zhang et al. reported that CP significantly reduced cancer stem cell (CSC) content, defined as aldehyde dehydrogenase (ALDH) over-expressed cells, in human breast, endometrial, or pancreas carcinoma cell lines *in vitro* [22]. CSCs were identified in various tumors including MM. Immature populations of cells such as CD138-negative cells and side populations have generally been accepted to have characteristics of CSCs in MM. Matsui et al. reported that CD138-negative MM cells exhibited significantly higher levels of ALDH activity than CD138-positive cells [23]. Additionally, the high drug resistance of CSC through the over-expression of ALDH is thought to be one of the major causes of drug resistance in MM [24]. In this study, we did not verify the effects of CP on the immature populations in MM cells; however, it may be possible that CP reduced CSC content including CD138-negative or ALDH over-expressed cells, as with solid cancers.

MM patients are exposed to many drugs with diverse mechanisms of action; these include bortezomib (BTZ), CFZ, and iMIDs. Unfortunately, the durations of such treatments are often prolonged [9], and this can lead to cross-resistance to multiple chemotherapeutic agents in RRMM patients. Thus, the challenge is to find new agents to treat RRMM individuals, and compounds that modulate ROS levels may be viable candidates in this regard. Two strategies to modulate ROS in MM for therapeutic benefits can be envisaged. The first is to inhibit ROS production by overexpressing antioxidant molecules or by treatment with antioxidants [5, 25]. Actually, we previously demonstrated that suppression of ROS production led to apoptosis in MM cells [5]. The second is to flood the cell with high levels of ROS, which cannot be buffered by endogenous antioxidant systems, as this triggers apoptosis in MM cells [26–29]. While MM is not currently treated with drugs that induce ROS as their primary mode of action, some reports in preclinical settings suggest that induction of oxidative stress is a promising treatment strategy [30, 31]. Indeed, oxidative stress is an important mechanism of cytotoxicity caused by proteasome inhibitors, including BTZ and CFZ [7, 8, 29]. In the present study, we found that CP synergized with CFZ or SAHA in MM cells, providing a framework for further studies of CP alone and in combination with other classes of drugs to improve the prognosis for MM patients.

In summary, we have identified that CP induces MM cell apoptosis through ROS over-production and depolarization of the mitochondrial membrane potential in a p53-independent fashion. Our results suggest a potential clinical application of CP for RRMM patients harboring p53 abnormalities, as these individuals generally respond poorly to currently approved MM therapeutics.

## MATERIALS AND METHODS

### Reagents and human cell lines

CP was obtained from Santa Cruz Biotechnology (Dallas, TX). Stock solutions of CP were generated by dissolving the powder in 100% dimethyl sulfoxide (DMSO; Sigma-Aldrich St. Louis, MO) at 10 mM. MM1S, RPMI8226, and U266 cell lines were kindly provided by Dr. Ruben D. Carrasco (Dana-Farber Cancer Institute, Harvard Medical School, Boston, MA). KMS11 and KMS5 were purchased from JCRB Cell Bank (Osaka, Japan). H929 cells were purchased from ATCC (Manassas, VA). All MM cell lines were cultured in RPMI 1640 (Sigma-Aldrich) containing 10% fetal bovine serum (FBS), 2 μM L-glutamine, and 1% penicillin-streptomycin. Bone marrow stromal cells (BMSCs) were cultured in DMEM (Sigma-Aldrich) supplemented with 10% FBS, 2 μM L-glutamine, and 1% penicillin-streptomycin.

### Patient specimens

Bone marrow specimens were obtained from patients with MM in accordance with Sapporo Medical University School of Medicine Review Board approval and informed consent was obtained in compliance with the Declaration of Helsinki. Primary CD138+ plasma cells were purified by positive selection with anti-CD138 magnetic-activated cell separation microbeads (Miltenyi Biotec, Auburn, CA) as described [32]. Primary BMSCs were established from CD138- bone marrow mononuclear cells as reported previously [33].

### Growth inhibition and apoptosis assays

MM cells (5 x10^3^) were treated for 48 h with CP at the indicated concentrations in 96-well plates. Subsequently, the inhibitory effect of CP on MM cell line growth was assessed using a WST-1 assay (Premix WST-1 Cell Proliferation Assay, Takara Bio, Otsu, Japan) and an Infinite M1000 PRO microplate reader (Tecan Japan, Kawasaki, Japan) as described previously [34]. The half-maximal inhibitory concentration (IC_50_) was defined as the drug concentration resulting in 50% cell survival relative to that of DMSO-treated cells. To analyze the proliferation of MM cells with or without BMSCs, we used a BrdU assay (BrdU cell proliferation assay reagent, Cell Signaling Technology, Danvers, MA) [5]. Initially, this assay was established using BrdU incorporated into cellular DNA during cell proliferation, and it is similar to the ^3^H-incorporation assay but does not use radioactive reagents. Therefore, the BrdU assay is suitable for this proliferation assay.

Apoptosis was evaluated by PARP, caspase-3, caspase-8, and caspase-9 western blotting and quantified using an Annexin V/7-AAD staining kit (BD Biosciences, San Jose, CA), as per the manufacturer’s instructions, followed by analysis on a BD FACS Canto II using FACSDiva (BD Biosciences, Tokyo, Japan) [35].

### Immunoblotting

MM cells were cultured with or without stimuli; cells were then harvested, washed, lysed, and stained using the following primary antibodies: PARP (#9542), p21 (#2947), caspase-3 (#9662), caspase-8 (#9746), caspase-9 (#9502), PUMA (#4976), and Bax (2772) (Cell Signaling Technology, Danvers, MA); and MDM2 (sc-965), p53 (sc-126), and actin-HRP (sc-1615) (Santa Cruz Biotechnology). Standard chemiluminescence was used to evaluate protein expression.

### RNA extraction and PCR arrays

RNA was extracted with TRIzol Reagent (Life Technologies, Carlsbad, CA) according to the manufacturer’s protocol. Total RNA (1 μg) was reverse transcribed using RT^2^ First Strand Kit (QIAGEN, Hilden, Germany). PCR-array was performed using RT^2^ Profiler PCR Array Human Oxidative Stress (PAHS-65Z) per the manufacturer’s protocol.

### Inhibition of p53 expression by small-interfering RNA (siRNA)

Non-silencing control siRNA (siRandom; #4390843, Thermo Scientific, Waltham, MA) and an siRNA targeting human p53 (sip53; sc-29453, Santa Cruz Biotechnology) were transfected into cell lines by nucleofection according to a previous report [36]. siRNA-transfected MM cells were incubated for 24 h and treated with CP for 24-48 h. Then cell proliferation or apoptosis was measured using a WST-1 assay or an Annexin V/7AAD assay.

### Measurement of ROS

Measurements of cytosolic or mitochondrial ROS were performed with CellROX Deep Red or MitoSOX Red (Invitrogen, Carlsbad, CA) according to the manufacturer’s protocol. Briefly, cells were stained for 10–30 min at 37°C with CellROX or MitoSOX. After staining, cells were washed and suspended in PBS. For quantification of intracellular or mitochondrial ROS, cells were analyzed with a BD FACS Canto II instrument. For fluorescence microscopic observation, cells were stained for 30 min at 37°C with 5 μM CellROX, 5 μM MitoSOX, 500 nM MitoTracker Green (Invitrogen), or 1 μg/ml Hoechst 33342 (Life Technologies, Carlsbad, CA). After staining, cells were washed and observed by live cell imaging using a Biozero BZ-8000 fluorescence microscope (KEYENCE Laboratories, Osaka, Japan).

### Mitochondrial membrane potential assay

Loss of mitochondrial membrane potential (Δψm) was quantified by tetramethylrhodamine, ethyl ester (TMRE, Invitrogen). MM cells (2 × 10^5^) were treated with CP for 0–24 h and cells were then collected and stained with 25 nM of TMRE for 30 min. Thereafter, fluorescence was measured using flow cytometry. Cells treated with 10 μM of FCCP (Sigma) were used as the positive control for depolarization.

### Murine xenograft model of human MM

NOD/Shi-scid IL-2γnul (NOG) mice (8-week-old females) were purchased from the Central Institute for Experimental Animals (Kawasaki, Japan). The animal studies were conducted according to protocols approved by the Animal Ethics Committee of the Sappro Medical University School of Medicine. Mice were inoculated subcutaneously in the left flank with 3 × 10^7^ RPMI8226 cells in 100 μl RPMI1640 with 100 μl Matrigel (Corning, Corning, NY). We planned the number of injected cells for the *in vivo* experiment according to previous reports [5, 37]. When the tumor volumes reached 100 mm^3^, mice were assigned to two groups receiving either the vehicle alone or CP. Each group of mice (n = 5) received vehicle only or CP (2 mg/mouse) suspended in vehicle (100 μl PBS), administered by intraperitoneal injection 3 days per week for 4 weeks [15]. Caliper measurements of the longest perpendicular tumor diameters were performed every other day to estimate the tumor volume using the following formula: length × width^2^ × 0.5. Complete necropsies were performed for each experimental animal. Isolated tissues were subjected to hematoxylin and eosin staining and immunohistochemical analysis with anti-cleaved PARP antibody (#5625 Cell Signaling Technology).

### Statistical analysis

Statistical significance of differences was determined using the Student *t* test. Statistical significance was defined at a value *P* < .01. IC_50_ values and combination index were calculated using CompuSyn software [38].

## SUPPLEMENTARY MATERIALS FIGURES


