# Supplementary material for: Small molecule CP-31398 induces reactive oxygen species-dependent apoptosis in human multiple myeloma
Source: Oncotarget. 2017 Jul 22;8(39):65889–99. doi: 10.18632/oncotarget.19508 (PMC5630380; doi:10.18632/oncotarget.19508)
Supplement: Supplementary file 1 [file oncotarget-08-65889-s001.pdf]

Small molecule CP-31398 induces reactive oxygen species-dependent apoptosis in human multiple myeloma

SUPPLEMENTARY MATERIALS

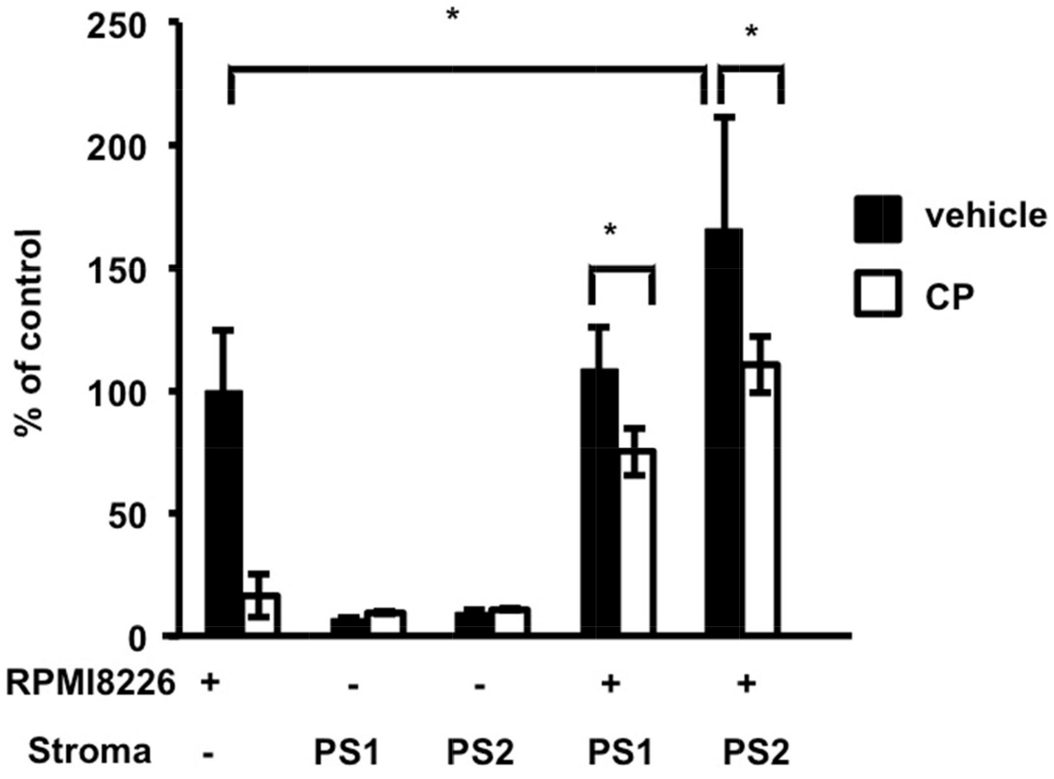

Supplementary Figure 1: CP maintained its anti-myeloma activity even in the presence of BMSCs. MM cells were incubated with CP (7.5 μM) in the absence (-) or presence (+) of BMSCs of two different MM patients (PS1, PS2). Cell viability was calculated using a BrdU assay at 48 h. We define the BrdU incorporation of "RPMI8226 (+), Stroma (-) group" as the control of this assay. Mt.: Mutated, WT: wild type. Data represent means of 6 independent cultures. Error bars represent the SD. *P< 0.01.

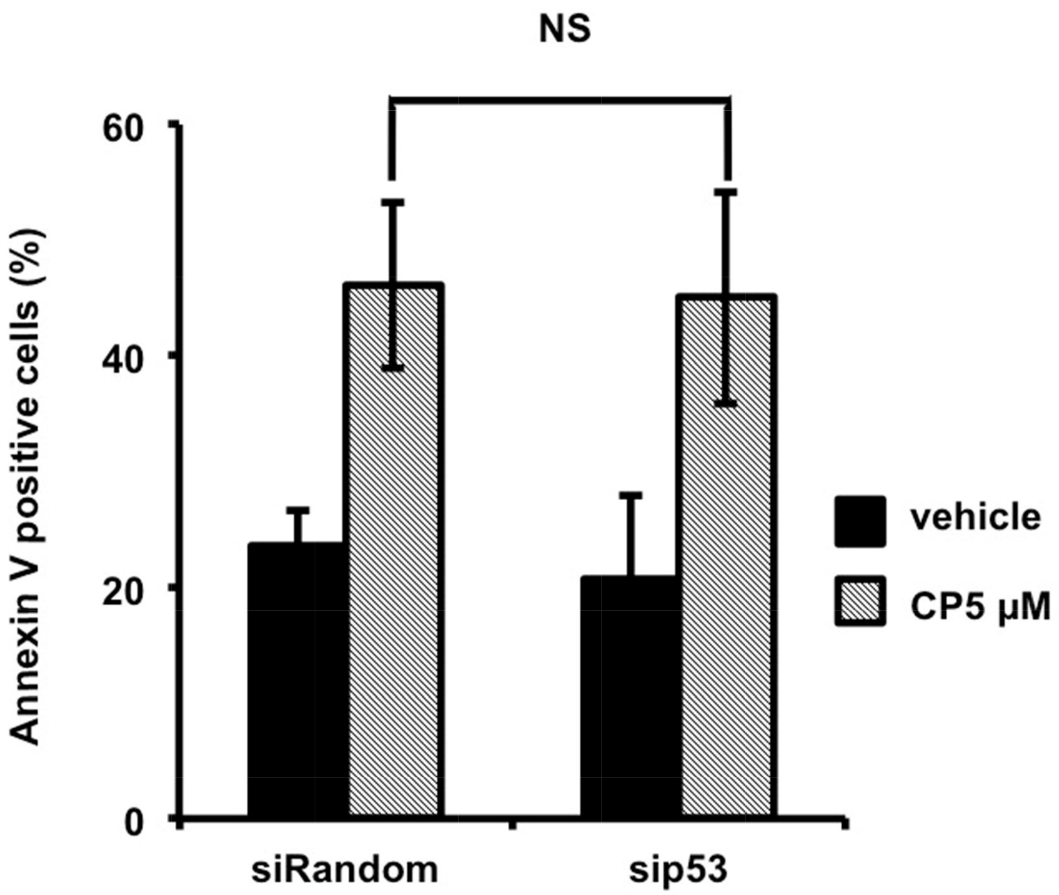

Supplementary Figure 2: p53-silenced RPMI8226 cells remain sensitive to CP. siRandom or sip53 transfected RPMI8226 cells were incubated with CP for 24 h and apoptosis was assessed using Annexin V/7AAD staining. Data are the mean of triplicate measurements. Error bars represent the SD. NS: not significant.

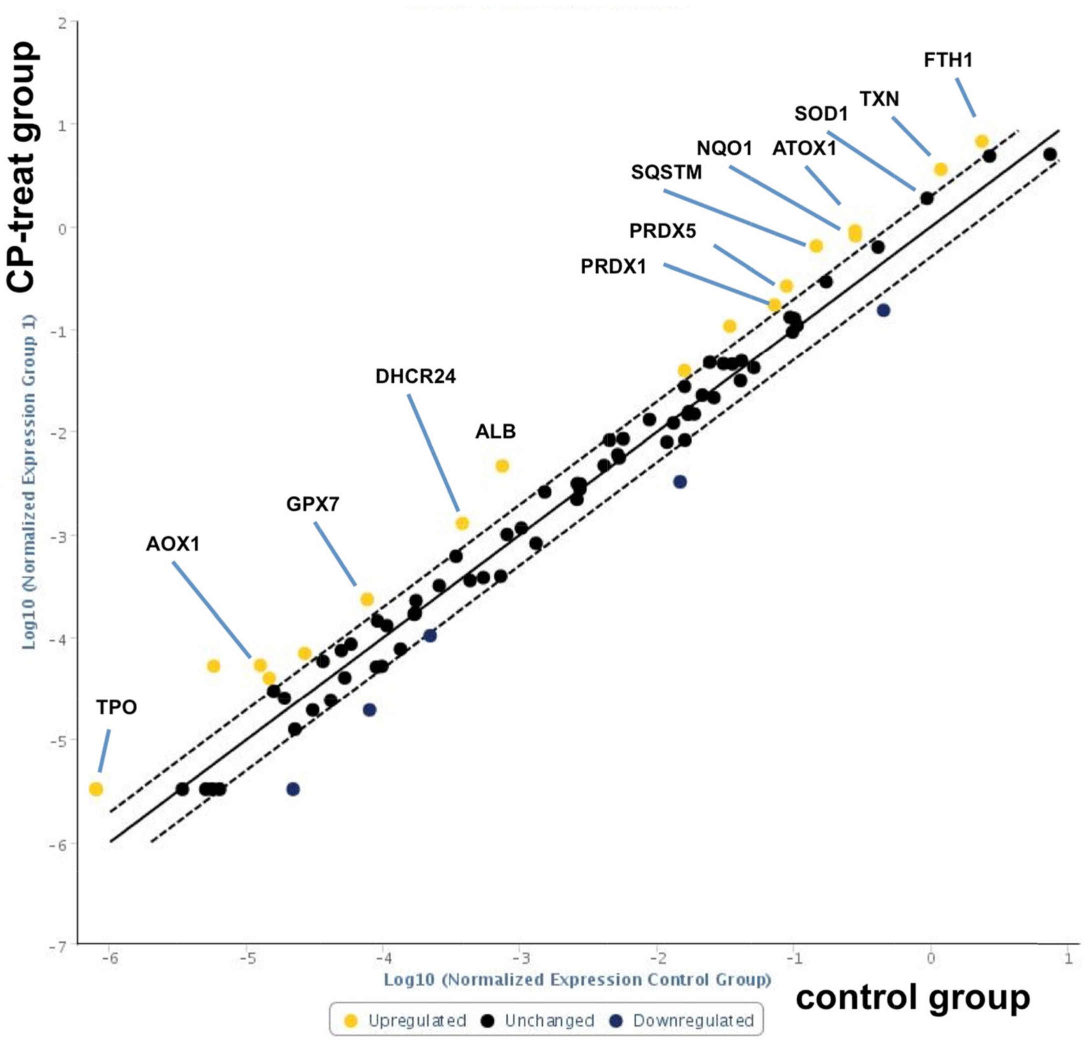

Supplementary Figure 3: CP induces different changes in the expression of oxidative stress-related genes. RNA from KMS11 (p53-null) cells treated with CP was characterized, and the results were compared to that of a vehicle (DMSO) control. CP induced different changes in the expression of oxidative stress-related genes than shown in the control group.

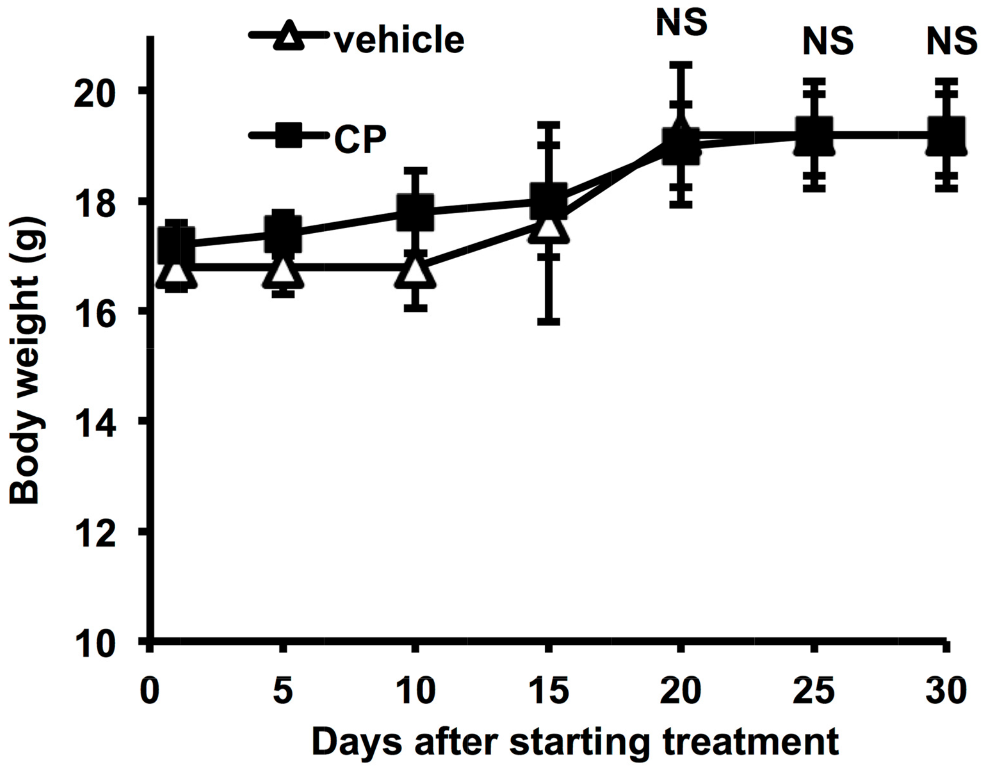

Supplementary Figure 4: Body weight changes in CP-treated mice. Body weight of mice treated with CP or vehicle at various time points after start of treatment. Data, mean ± S.D. NS: not significant.

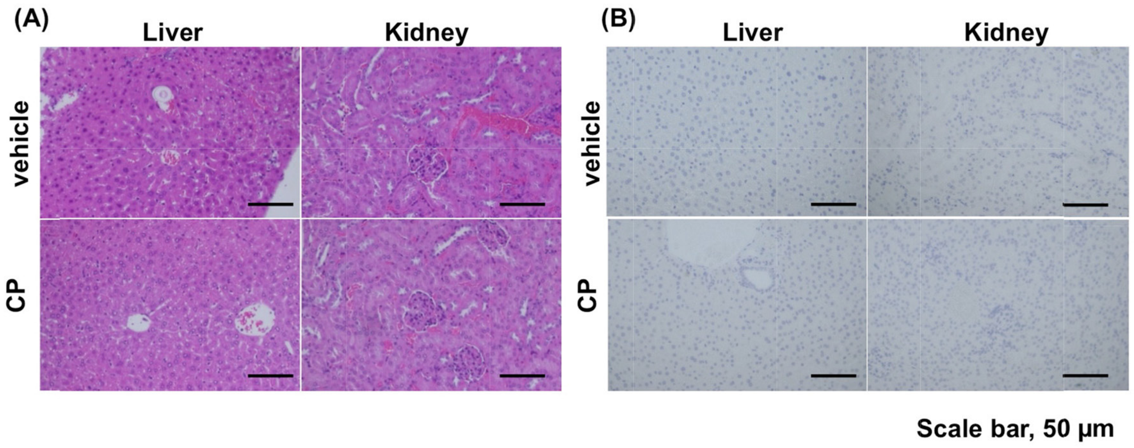

Supplementary Figure 5: Histology of liver and kidney of CP-treated mice. Histologic analysis of liver and kidney isolated from experimental mice stained with **(A)** hematoxylin and eosin (H&E) and **(B)** cleaved-PARP antibody isolated from vehicle or CP-treated mice. Scale bars, 50 μm. *$P < 0.01$.

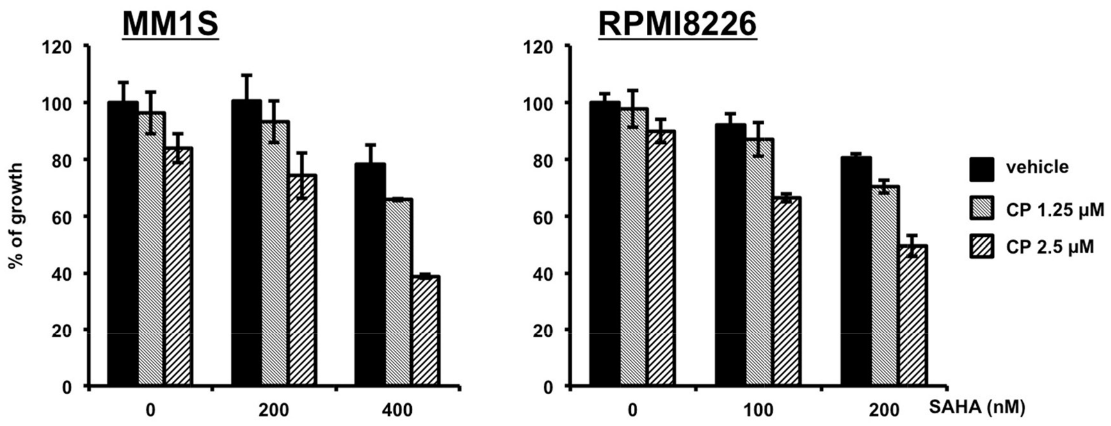

Combination Index

MM1S:

		SAHA (nM)	
		200	400
CP (μM)	1.25	0.84	0.41
	2.5	0.91	0.57

RPMI8226:

		SAHA (nM)	
		100	200
CP (μM)	1.25	1.13	0.98
	2.5	0.84	0.78

Supplementary Figure 6: CP combined with SAHA inhibits the proliferation of cultured MM cells synergistically. MM1S or RPMI8226 cells were incubated 48 h with CP and SAHA. Thereafter, cell viability was assessed using a WST-1 assay. Data represent means of 6 independent cultures. Error bars represent SD. Combination Index, CI. CI <1, synergetic; CI=1, additive effect.
